# Photoprotective Strategies of Mediterranean Plants in Relation to Morphological Traits and Natural Environmental Pressure: A Meta-Analytical Approach

**DOI:** 10.3389/fpls.2017.01051

**Published:** 2017-06-19

**Authors:** Beatriz Fernández-Marín, Antonio Hernández, Jose I. Garcia-Plazaola, Raquel Esteban, Fátima Míguez, Unai Artetxe, Maria T. Gómez-Sagasti

**Affiliations:** Department of Plant Biology and Ecology, University of the Basque Country (UPV/EHU)Bilbao, Spain

**Keywords:** antioxidant, carotenoid, evergreen, Mediterranean, photoprotection, photosynthetic pigment, sclerophyllous, tocopherol

## Abstract

Despite being a small geographic extension, Mediterranean Basin is characterized by an exceptional plant biodiversity. Adaptive responses of this biocoenosis are delineated by an unusual temporal dissociation along the year between optimal temperature for growth and water availability. This fact generates the combination of two environmental stress factors: a period of summer drought, variable in length and intensity, and the occurrence of mild to cold winters. Both abiotic factors, trigger the generation of (photo)oxidative stress and plants orchestrate an arsenal of structural, physiological, biochemical, and molecular mechanisms to withstand such environmental injuries. In the last two decades an important effort has been made to characterize the adaptive morphological and ecophysiological traits behind plant survival strategies with an eye to predict how they will respond to future climatic changes. In the present work, we have compiled data from 89 studies following a meta-analytical approach with the aim of assessing the composition and plasticity of photosynthetic pigments and low-molecular-weight antioxidants (tocopherols, glutathione, and ascorbic acid) of wild Mediterranean plant species. The influence of internal plant and leaf factors on such composition together with the stress responsiveness, were also analyzed. This approach enabled to obtain data from 73 species of the Mediterranean flora, with the genus *Quercus* being the most frequently studied. Main highlights of present analysis are: (i) sort of photoprotective mechanisms do not differ between Mediterranean plants and other floras but they show higher plasticity indexes; (ii) α−tocopherol among the antioxidants and violaxanthin-cycle pigments show the highest responsiveness to environmental factors; (iii) both winter and drought stresses induce overnight retention of de-epoxidised violaxanthin-cycle pigments; (iv) this retention correlates with depressions of Fv/Fm; and (v) contrary to what could be expected, mature leaves showed higher accumulation of hydrophilic antioxidants than young leaves, and sclerophyllous leaves higher biochemical photoprotective demand than membranous leaves. In a global climatic change scenario, the plasticity of their photoprotective mechanisms will likely benefit Mediterranean species against oceanic ones. Nevertheless, deep research of ecoregions other than the Mediterranean Basin will be needed to fully understand photoprotection strategies of this extremely biodiverse floristic biome: the Mediterranean ecosystem.

## Introduction

Since late-90s, Mediterranean biome is recognized as a biodiversity hotspot and a global conservation priority (Cox and Underwood, 2011; Bellard et al., 2014; Matesanz and Valladares, 2014). It should be underlined that the biodiversity maintenance is essential to ecosystem health and resilience and, consequently, the services that they provide (Pereira et al., 2013). Accordingly, important efforts and resources are being invested in the study and preservation of the exceptional diversity of the Mediterranean vascular flora that includes many rare and endemic taxa (Cox and Underwood, 2011; Bellard et al., 2014). Despite these efforts, the loss of biodiversity in the Mediterranean biome does not seem to slow down (Butchart et al., 2010). Anthropogenic activities such as intensive agriculture, fire-regimes, grazing, and urbanization have strongly altered Mediterranean ecosystems, especially in the last decades (Bellard et al., 2014; Matesanz and Valladares, 2014), having negative consequences into native plant communities (Hoekstra et al., 2004; Cox and Underwood, 2011). As the world population continues to grow, the extension of natural habitats in this diminutive biome will probably continue to reduce (Cox and Underwood, 2011).

Moreover, the future holds new threats: Mediterranean regions are expected to be heavily impacted by climate change (Klausmeyer and Shaw, 2009). Climatic scenarios point out not only an increase of aridity but also a higher variability and frequency of extreme climatic events, such as heat waves and droughts, particularly in the Mediterranean areas of Southern and Western Europe (Jacob et al., 2014; Matesanz and Valladares, 2014). The expected ecological effects on vegetation include changes in life cycles (Gordo and Sanz, 2009) and mortality (Gitlin et al., 2006), invasions and/or attacks of pathogens and changes in species composition (Aitken et al., 2008; Hickler et al., 2012; Bussotti et al., 2015), among others. Hence, the study of functional and morphological traits of native Mediterranean species in response to environmental stresses is of paramount importance to delineate future affections and lines of action.

In essence, Mediterranean plant biodiversity is mainly configured by the peculiar climatic drivers of the region (read seasonality) characterized by a temporal dissociation between optimal temperature and availability of water needed for growth. Summers are characterized by a drought period of variable length, intensive sunshine and moderately to extremely high temperatures, whilst winters are mild to cold with variable rainfall. The co-occurrence of light excess with drought in summer and with low temperatures in winter makes both periods the most stressful for vegetation in Mediterranean environments (Mitrakos, 1982). Thus, under these conditions and provoked by the imbalance between light capture and use, plants may suffer photo-oxidative damage of photosystem II (PSII) due to their inability to use the excess of absorbed light (Murata et al., 2007). Photoinhibition, beyond the photodamage of proteins of PSII such as D1 protein, is associated with the impairment of various photoprotective processes that ensure the integrity of the PSII complex, including thermal dissipation of excitation energy (Ort, 2001; Barker et al., 2002). Therefore and depending on the degree of plasticity (i.e., ability to acclimate -and possibly adapt- to different environmental conditions), Mediterranean plants display a suite of morpho-anatomical, physiological and biochemical adjustments to avoid photooxidation and photodamage (Osmond et al., 1997).

Numerous studies have provided evidence of the capacity of Mediterranean plants to adjust their morphology, physiology, phenology and reproduction in response to varying temperature, nutrients, light, and water availability (Domínguez et al., 2012; Matesanz and Valladares, 2014 and references therein). Some of the most common and representative traits of Mediterranean plants include the small size and bushy phenotypes with short internodes, the small and thick leaves: usually sclerophyllous and evergreen (long life span) with high Leaf Mass per Area (LMA) in agreement with leaf economics spectrum (Wright et al., 2004), and the presence of abaxial trichomes. All these traits relate to the generally high water use efficiency (WUE) typical of Mediterranean plants, that is achieved through a complex interaction of mechanisms enabling a reduction of water loss per unit of carbon gain (Matesanz and Valladares, 2014). Yet, an integrated overview of the interplay between these physical traits of Mediterranean plants and their photoprotective mechanisms at metabolic level is still missing.

At biochemical level, is known that plants generally counteract photo-oxidative damage through fine-tuning of a variety of photoprotective mechanisms based on specialized metabolites that keep reactive oxygen species (ROS) at a concentration efficient for signaling (Hadacek and Chobot, 2011). Such mechanisms include: (1) decrease of total chlorophyll (Chl a+b) content and increase of the ratio Chl a/b; (2) activation of an intricate antioxidant network that includes, for instance, the synthesis of non-enzymatic low molecular weight hydrophilic antioxidants such as glutathione (tGSH) and ascorbic acid (Asc), or the accumulation of lipophilic antioxidants such as α-tocopherol (α-Toc) and β-carotene (β-Car); and (3) conversion of the xanthophylls from the violaxanthin (VAZ-cycle) and the lutein-epoxide cycle (LxL-cycle) toward de-poxidised forms: antheraxanthin (Ant), Zeaxanthin (Zea), and Lutein (Lut) (Valladares et al., 2012). Ubiquitous VAZ-cycle, and uncommon LxL-cycle are both involved in the process of thermal dissipation of excess light to PSII through non-photochemical quenching (NPQ) (García-Plazaola et al., 2007; Esteban and García-Plazaola, 2014; Esteban et al., 2015a). Additionally, Zea and β-Car as well as α-Toc operate not only as physical and chemical quenchers of singlet oxygen (^1^O_2_) but they can also modify membrane fluidity (Munné-Bosch et al., 2013; Di Ferdinando et al., 2014), offering a double protection: they prevent the formation of Chl triplet excited state and protect the integrity of chloroplast. Concomitantly, tAsc and tGSH are intimately related to ROS detoxification and, particularly, hydrogen peroxide removal (Szarka et al., 2012). Above-mentioned photoprotective pigments and antioxidants comprise a complex network. It has long been known that Asc and GSH form ascorbate-glutathione cycle, where Asc is regenerated by GSH (Foyer and Noctor, 2011). Ascorbic acid additionally acts as a cofactor for Vio de-epoxidation to Zea in the VAZ-cycle and is also involved in the regeneration of α-Toc (Venkatesh and Park, 2014). Quantitative changes in the profile of previously detailed metabolites, which could be understood as “antioxidant plasticity,” frequently reflect deeper structural and functional modifications of the photosynthetic apparatus (Esteban et al., 2015a). Nonetheless, it is important to identify plant functional traits in which plasticity may play a determinant role not just in plant survival under strong seasonal climates such as the Mediterranean climate, but also in predicted novel environmental conditions (Gratani, 2014). Independent studies have characterized photoprotective strategies of Mediterranean species from different natural locations along the last two decades (e.g., García-Plazaola et al., 1999, 2008; Munné-Bosch et al., 2003; Hormaetxe et al., 2007; etc.). Nevertheless, the remarkably high diversity of plants in this ecosystem and the complexity of the photoprotective and antioxidant mechanisms (i.e., accumulation of antioxidants is generally triggered under slight to moderate stress but some are consumed and thus their content decrease because of extenuation under extreme stress) point out the need of a comprehensive compilation of data available that could enhance our understanding of Mediterranean plant photoprotective responses, estimation of their plasticity indexes and prediction of their fitness in a scenario of climate change.

Recently, morpho-physiological traits of Mediterranean plats have been compiled and deeply analyzed (e.g., Domínguez et al., 2012; Niinemets, 2015; etc.). Nevertheless, comparable studies to ascertain the biochemical traits in terms of photoprotective pigments and antioxidants are much scarcer and there is lack of a joined synopsis combining both morphological and biochemical photoprotective traits. Here, we present a literature compilation focused on the photoprotection of wild species from the Mediterranean Basin to add light to this topic. Specially we aimed at (i) providing comprehensive and quantitative synthesis of reference values of photoprotective pigments and antioxidant of native Mediterranean plant species living under non-stressed conditions; (ii) elucidating how these biochemical traits relate to morphological and functional leaf and plant traits; and (iii) assessing how they change under the abiotic stresses associated to Mediterranean climate (“antioxidant plasticity”).

## Materials and methods

### Literature search

Data were compiled from peer-reviewed journals only, using the “ISI Web of Sciences” source. Only original articles written in English and published in the last 20 years, covering the time laps between January 1996 and May 2016, were considered. Key words used in the search included (i) antioxidant-related terms: “tocopherol OR glutathione OR ascorb^*^ OR zeaxanthin OR lutein OR caroten^*^ OR antioxidant OR ^*^oxid^*^,” (ii) Mediterranean ecosystems-related terms “Mediterranean OR scrub OR chaparral OR shrubland OR woodland OR *Quercus* OR *Arbutus* OR *Cistus* OR *Rosmarinus* OR *Pistacia* OR *Lavandula*),” and (iii) terms related to photosynthetic tissues: “leaf OR leaves OR foliar OR chloroplast^*^.” The search was also filtered by areas of knowledge: “Plant Sciences OR Agriculture OR Environmental Sciences Ecology OR Forestry.” A total number of 840 articles fitted to these criteria.

### Manuscripts inclusion criteria

From the initial search, manuscripts were partitioned according to the following principles: only wild species original from the Mediterranean bioclimatic region were included. Field studies with Mediterranean species conducted out of the Mediterranean bioclimatic region or with aquatic plants (i.e., *Posidonia*), or with crops, were excluded. Works performed under growth chamber or greenhouse conditions were excluded. Finally, only papers using high-performance liquid chromatography for carotenoid and/or tocopherol quantification were included. After all these criteria our final database comprised data from 89 papers, 73 species, and 32 families (Table S1).

### Input data for the analysis

Numeric data were extracted from text, tables and figures of the 89 selected manuscripts (Table S2). Data from figures were obtained by using the “scale tool” from the software Adobe PhotoShop, CS5 Extended, v 12.1 ×64. Different type of numeric data were obtained from the manuscripts: (1) *foliar content of photosynthetic pigments and small molecular weight antioxidants:* anteraxanthin (Ant), neoxanthin (Neo), violaxanthin (Vio), lutein (Lut), lutein epoxide (Lx), zeaxanthin (Zea), chlorophyll a (Chl a), chorophyll b (Chl b), β-carotene (β-Car), α-tocopherol (α-Toc), total ascorbate (tAsc), total glutathione (tGSH) and/or sum or ratios of these parameters i.e., (A+Z)/(V+A+Z) (so far referred to as AZ/VAZ), Chl a+b, pigments or antioxidants per Chl, etc.; (2) *parameters indicative of stress:* maximal photochemical efficiency of PSII (Fv/Fm), predawn water potential (Ψ), relative water content (RWC), midday stomatal conductance (g_s_); (3) *parameters relating leaf mass and area*: leaf mass per area (LMA), specific leaf area (SLA). In the case of photosynthetic pigments and antioxidants, values expressed per fresh weight (FW), dry weight (DW), leaf area or Chl content were obtained. When any of these data was missing, and whenever possible, they were calculated from the original data from the paper. In some cases, units were converted.

Further details collected from each paper regarded *plant developmental stage* (seedling, adult), *leaf age* (young, mature), *time of the day* (predawn, artificial predawn (> 12 h darkness), morning, midday, evening). Additionally, information about *growth type* (woody: tree, shrub; non-woody: herb), *taxonomic group* (angiosperm, gymnosperm), *leaf texture* (membranous, sclerophyllous, malacophyllous, succulent), *leaf attributes* (none, waxy, pubescent, hairy-abaxial) and *leaf phenology* (evergreen, winter-deciduous, summer-deciduous) were compiled from the available literature.

### Data processing

**Figure 1:**
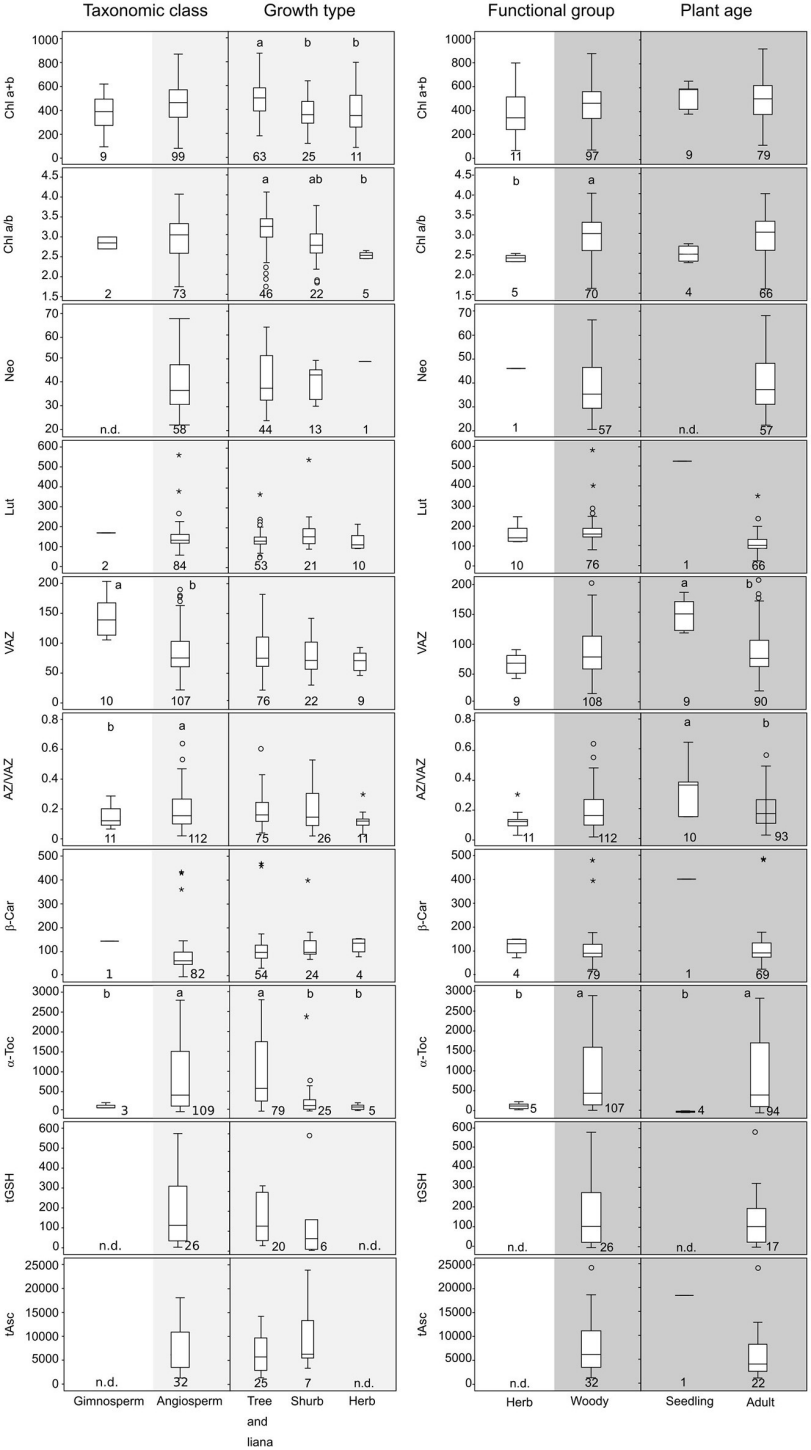
Boxplots showing the concentrations of the main photosynthetic pigments grouped considering different plant traits. **(Left)** Taxonomic class (Gymnosperm vs. Angiosperm) and growth type (Tree and liana, Shrub, and Herb) within angiosperms. **(Right)** Functional group (life style) (Herb vs. Woody) and plant age (Seedling and Adult) within woody. Total chlorophyll (Chl a+b) is expressed in μmol m^−2^. Carotenoids such as neoxanthin (Neo); lutein (Lut); pool of violaxanthin, anteraxanthin and zexanthin (VAZ); β-carotene (β-Car) and α-tocopherol (α-Toc) are expressed in mmol mol^−1^ of total Chl. Hydrophilic antioxidants such as glutathione (tGSH) and ascorbic acid (tAsc) are also expressed in mmol mol^−1^ of total Chl. De-epoxidation state of violaxanthin cycle (AZ/VAZ) is expressed in mol mol^−1^; Only data for non-stressed plants are included. Boxes cover 50% of the data; central lines represent the medians and whiskers represent the minimum and maximum values among non-atypical data. Open circles and asterisk represent outliers and extreme outliers, respectively. The number of reported data (*n*) is shown below each box. Lowercase and upper case letters above the boxes denote significant differences (*P* < 0.05) among groups analyzed by one-way ANOVA (normally distributed data) or by Kruskal-Wallis ANOVA (non-normally distributed data).

**Figure 2:**
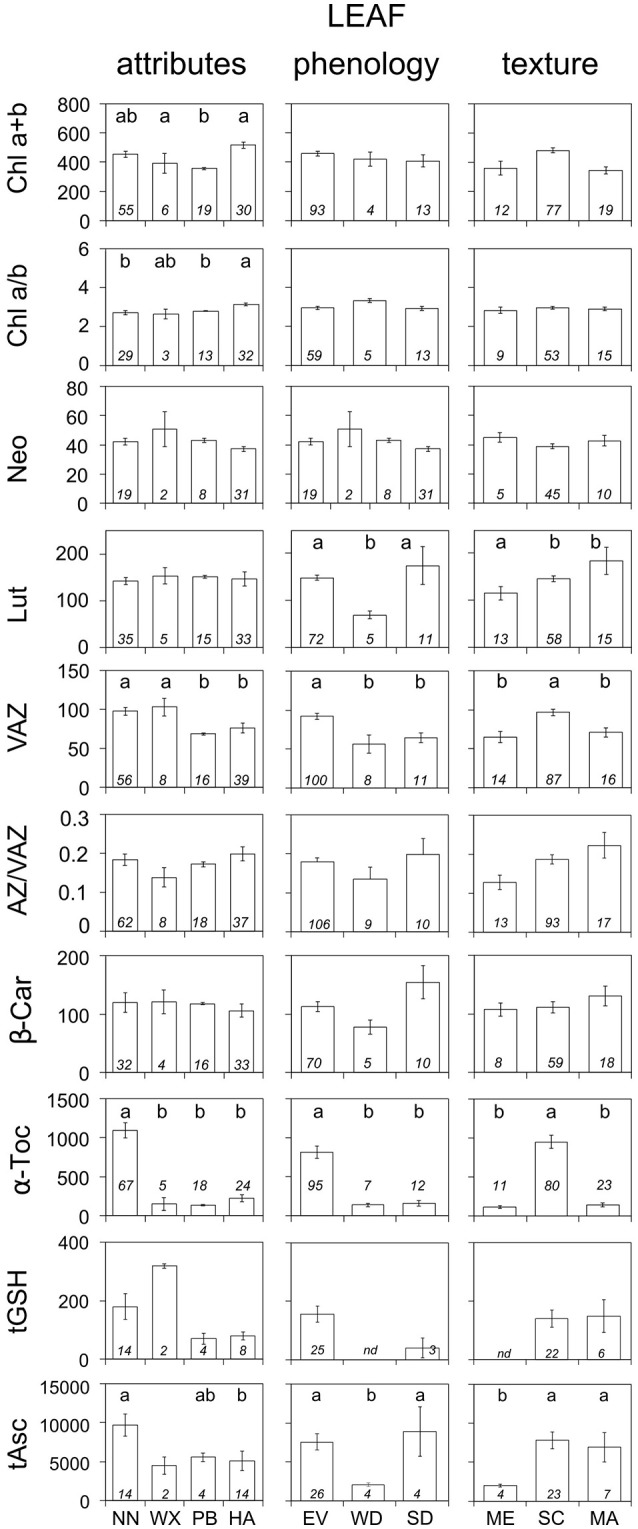
Average composition of photosynthetic pigments and antioxidants according to different leaf traits: attributes, phenology and texture. Units are as in Figure 1. Each bar represents the average ± SE of available data (*n*, shown inside the bars). Only data for non-stressed plants were included. When significant, differences in the content of pigments or antioxidants among categories inside each panel are depicted with letters above the bars (*P* < 0.05). “nd” depicts no data available. Abbreviations as in Table 2.

**Figure 3:**
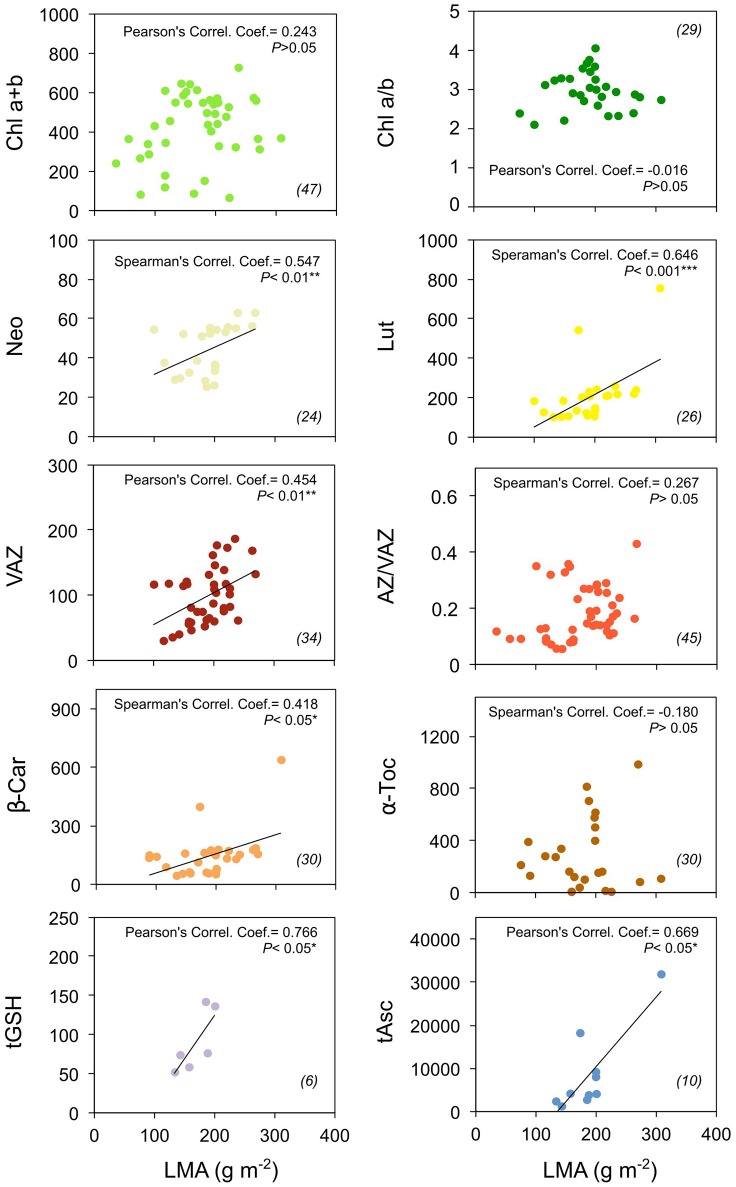
Photosynthetic pigment and antioxidant contents in relation to leaf mass area (LMA) of “non-stressed” Mediterranean plants. Pearson's (or Spearman's for non-normally distributed data) correlation coefficient and significance, and number of data (in brackets) are shown for each panel. Units are as in Figure 1. Asterisks denote the extent of significance. Only predawn data were included for this analysis.

Every manuscript was first labeled with regard to its main stress or topic (i.e., drought, salinity, climate-comparison, high temperature, low temperature, species comparison, etc.). Secondly, each data-row within the manuscript was labeled as control or treatment. When a treatment was designated as “control” in the study, it was included as such in the database. Nevertheless, there were some manuscripts in which there were not real controls. In those cases, the less stressful condition was considered as control for each study. Apart from this division, and taking into consideration all the manuscripts together, data from non-treated plants, together with real controls, were considered as “non-stress” values (data were excluded from this category if their Fv/Fm values were < 0.6, predawn AZ/VAZ > 0.4 and/or predawn Ψ < −2 MPa). Data from “non-stress” category were used for the characterization of photosynthetic pigment and antioxidant composition in Mediterranean species (Table 1), and for the assessment of their composition according to plant biological traits (Figure 1), and leaf traits (Figures 2, 3, **6**).

**Table 1:** Composition of photosynthetic pigment and low molecular weight antioxidants in a representation of wild Mediterranean species (covering 9 different families).

	**Fam**.	**Anacardiaceae**	**Buxaceae**	**Caprifoliaceae**		**Cistaceae**			**Ericaceae**	**Fagaceae**			**Lamiaceae**		**Oleaceae**	**Rhamnaceae**
	**Sp**.	***Pistacia lentiscus***	***Buxus sempervirens***	***Lonicera implexa***	***Viburnum tinus***	***Cistus albidus***	***Cistus clusii***	***Cistus salviifolius***	***Arbutus unedo***	***Quercus coccifera***	***Quercus ilex***	***Quercus suber***	***Rosmarinus officinalis***	***Salvia officinalis***	***Phyllirea latifolia***	***Rhamnus alaternus***
Chl a+b	Mean	344	321	305	377	303	348	476	407	498	547	521	278		487	564
	Max	379	546	336	395	467	375	625	561	616	791	639	408		583	688
	Min	288	257	262	356	151	311	370	178	223	339	180	117		381	344
	*n*	5	7	3	3	5	3	4	5	15	18	8	3		3	3
Chl a/b	Mean	2.46	2.22			3.03	2.78			2.81	3.26	3.30	2.96	2.52		
	Max	2.94	3.72			3.73	2.85			3.45	4.07	3.51	3.12	2.60		
	Min	1.79	1.75			2.65	2.67			2.10	1.97	3.10	2.80	2.40		
	*n*	5	5			3	6			6	17	6	3	3		
Neo	Mean		36							45.8	35.9	33				
	Max		41							62.6	56.3	51				
	Min		32							29.0	25.3	24				
	*n*		3							6	17	7				
Lut	Mean		159			132	323	118	116	153	138	270	270			
	Max		188			186	543	120	124	210	218	412	412			
	Min		121			93	200	114	106	123	102	196	196			
	*n*		6			4	3	3	3	7	18	3	3			
VAZ	Mean	86	112	86	106	56		52	85	111	83.1	47	47		114	95
	Max	135	153	98	129	94		57	134	176	197	67	67		182	126
	Min	52	35	66	73	30		47	62	30	36	9	9		89.5	75
	*n*	3	7	3	3	4		3	4	16	24	3	3		4	3
AZ/VAZ	Mean	0.34	0.18	0.16	0.16	0.19	0.44	0.21	0.32	0.23	0.25	0.21	0.23	0.08	0.19	0.11
	Max	0.87	0.36	0.21	0.21	0.31	0.50	0.53	0.78	0.49	0.86	0.30	0.29	0.12	0.37	0.13
	Min	0.04	0.02	0.11	0.11	0.09	0.33	0.06	0.05	0.09	0.04	0.09	0.13	0.03	0.09	0.08
	*n*	5	7	3	3	4	4	4	6	17	26	7	3	3	4	4
β-Car	Mean		86			102	185	101	80	110	101	91	136			
	Max		97			121	397	122	100	173	175	161	218			
	Min		68			92	126	87	47	31	53	61	83			
	*n*		6			3	5	3	3	8	19	7	5			
α-Toc	Mean	1,599	1,534	260	745	259	153	195		469	304		52		288	323
	Max	2,798	3,914	414	1,442	433	344	286		606	815		156		416	496
	Min	7	69	63	220	96.4	36	89		212	1		4		109	207
	*n*	38	5	3	3	4	8	4		3	11		5		3	3
	Mean		680	318	301	134		7	37	30	85				127	323
tGSH	Max		789	326	321	158		9	48	31	141				140	328
	Min		571	310	280	109		5	26	28	34				115	318
	*n*		2	2	2	2		2	2	2	6				2	2
	Mean		23,339	4,609	8,041	4,056		7,390	13,459	12,090	5,762				8,836	9,084
tAsc	Max		24,025	5,730	9,694	4,801		8,545	14,258	12,102	13,553				12,950	9,336
	Min		22,654	3,488	6,388	3,310		6,236	12,660	12,079	1,335				4,723	8,832
	*n*		2	2	2	2		2	2	2	7				2	2

*Data are mean, maximum and minimum for each species and metabolite. Number of cases is designated as n. Species for which higher number of cases was available were selected: i.e., n ≥ 3 (or n ≥ 2 for hydrophilic antioxidants) for at least two metabolites. Units as in Figure 1*.

To evaluate the influence of the main Mediterranean climatic factors over photosynthetic pigments and antioxidants, manuscripts dealing with drought (D), low temperature (LT), high temperature (HT), and/or seasonality (S) were analyzed in detail (total manuscripts = 43). Within each manuscript, an index of variation with respect to the control was calculated for each of the climatic stressors independently. Hence, all data values were normalized to their respective controls. After data transformation, controls resulted as 1, while the deviation from the value 1 indicated the variation of the parameters analyzed. Reference values (controls) were considered the ones cataloged as “control” in the original papers.

To study the effect of leaf age on photosynthetic pigment and antioxidant content, only those studies where both mature and young leaves were simultaneously reported were included (total articles = 8). The young leaves were taken as reference values and the fold-changes were calculated as follows: Fold-change = (value of mature leave/value of young leave) −1. Thus, reference value was equaled to zero.

Plasticity Index (PI) for each parameter (Fv/Fm, pigments, antioxidants and LMA) was estimated according to Valladares et al. (2006) with the following formula: (Max-Min)/Max (within each paper of origin and parameter). This index generates values between 0 (no plasticity) and 1 (highest plasticity) comparable among parameters. A minimum of three different papers for each parameter was used as source of data for the calculation of average PI values. Only papers with two or more data for at least one species and one parameter where selected.

### Statistical analyses

**Figure 4:**
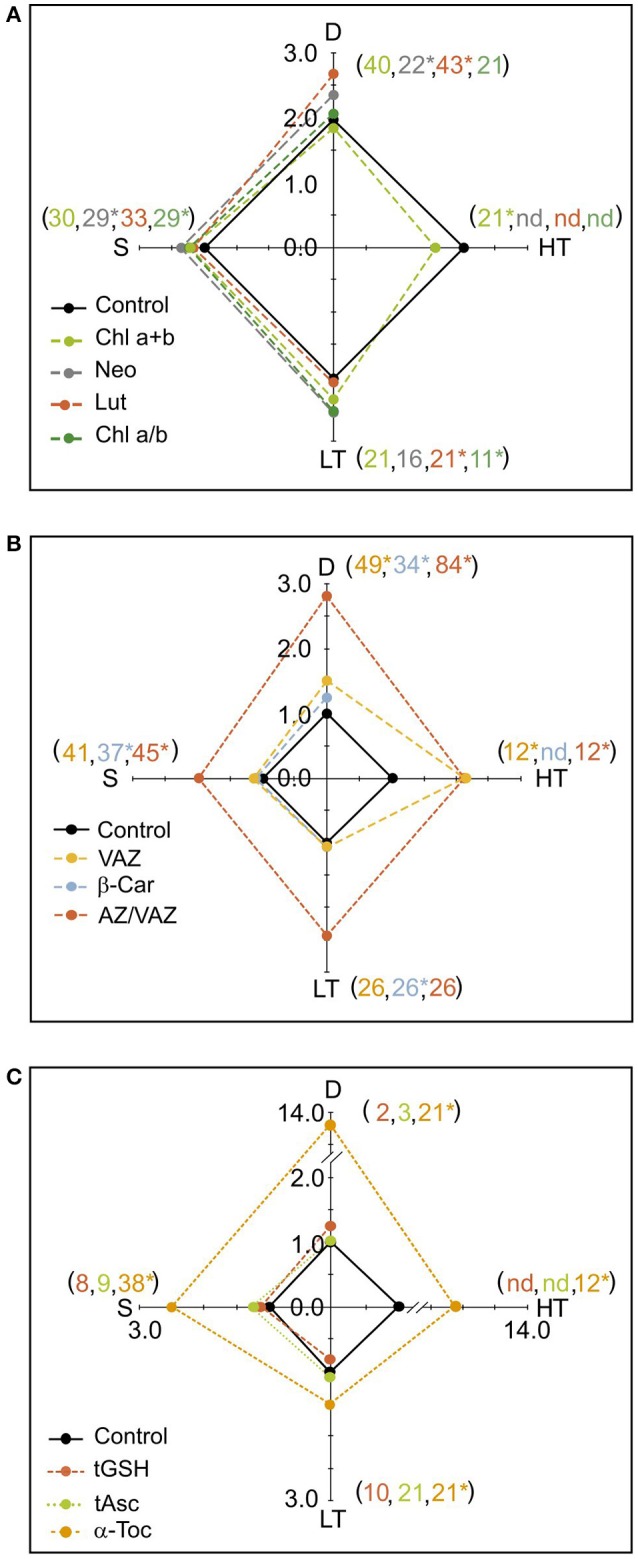
Spiderplots showing the effect of the main stress factors considered in the Mediterranean regions (D, drought; HT, high temperature; LT, low temperature; S; Seasonality) on photosynthetic pigments and antioxidants. **(A)** Photosynthetic pigments: chlorophylls and structural xanthophylls Neo and Lut. **(B)** Photosynthetic pigments: V-cycle xanthophyls and b-Car. **(C)** Free antioxidants: tGSH, tASC and a-Toc. Units are as in Figure 1, except for tGSH (here shown in μmol g^−1^ DW). For each stress factor, every data was normalized to its respective control within each manuscript of origin (controls were values cataloged as “control” in the original papers). Thus, value 1 indicates the control in the boxplots, while the deviation from 1 indicates the impact of the stress on the parameters analyzed. For more details see material and methods. Shown data are means of several cases. The number of cases used for each factor and compound is shown in brackets. Note that the color of the number indicates the corresponding metabolite. SE is not shown for clarity, but was <6% of the mean in all cases except from α-Toc, for which SE < 10%. Asterisks denote significant differences from control (*P* < 0.05).

One-way ANOVA (for normally distributed data) and a Kruskal-Wallis ANOVA (for non-normally distributed data) were applied to test for significant differences among categories within functional groups, growth forms and plant developmental stages (Figure 1). Non-parametric Mann Whitney-U test was used to check for significant differences in the content of antioxidants and pigments among different leaf traits (Figure 2). Pearson's correlation was used to check for relationship between LMA and photosynthetic pigments or antioxidants (Figure 3). Student's *t*-test was performed in order to assess significant differences between control and climatic stresses (Figure 4). Student's *t*-test (or single sample Kolmogorov-Smirnov-test, for non-normally distributed data) was performed to assess differences between young and mature leaves (**Figure 6**). The resulting *P*-values were considered to be statistically significant at α = 0.05. All statistical analyses were performed with SPSS 24.0 (IBM Corp., Armonk, NY, USA).

## Results

### Pigment and antioxidant composition of mediterranean species

Table 1 depicts reference values of photosynthetic pigments and antioxidants from a representative selection of native Mediterranean species living under non-stressful conditions (see Table S3 for an extended data set including parameters with *n* < 3). Average Chl a+b content was remarkably stable, fluctuating between 278 (μmol m^−2^) in *Rosmarinus officinalis*, and 547 in *Quercus ilex*, with the highest values being found in sclerophyllous species from Fagaceae and Rhamnaceae families. Among Fagaceae, sclerophyllous *Quercus* showed, additionally, the highest Chl a/b ratio. The content of α-Toc was the most variable among lipophilic antioxidants, varying up to two orders of magnitude among species (e.g., *Pistacia lentiscus* vs. *Rosmarinus officinalis*) and tGSH among hydrophilic antioxidants, varying also up to two orders of magnitude (e.g., *Cistus salviifolius* vs. *Buxus sempervirens*, Table 1). The intraspecific variability within a species (i.e., maximal amplitude between minimum and maximum values) was the highest for most of the metabolites in the case of *Quercus* species (except for the α-Toc, that appeared as extremely variable in *Pistacia lentiscus)*.

### Effect of internal plant factors

The internal plant factors considered in the present work were the taxonomic class, the functional group, the growth form and the plant age (Figure 1). Our analysis revealed that pigment and antioxidant content in Mediterranean wild plant species under non-stressed conditions was differentially distributed depending on taxonomic class (gymnosperm or angiosperm) and functional group (herbaceous or woody) (Figure 1). In this regard, we identified two critical gaps of knowledge that introduce uncertainty to data interpretation: the lack of information about photoprotective features in gymnosperms and in herbs. Even so, when gymnosperms and angiosperms were compared, significantly higher values of AZ/VAZ and α-Toc were observed in the latter (Figure 1, left). After subdividing this taxonomic class into growth types, significant differences were found for α-Toc, being much more abundant in trees and lianas than herbs. Total Chl and Chl a/b were also significantly higher in trees and lianas than in the rest of angiosperm groups.

In agreement with this, when the functional group (herbaceous vs. woody) of Mediterranean plants was considered (Figure 1, right), levels of Chl a/b and α-Toc were significantly higher in woody species. Within them, we also analyzed the effect of plant age on the antioxidant load. Interestingly, VAZ and AZ/VAZ decreased while α-Toc increased with plant age (Figure 1, right).

### Effect of leaf properties

The influence of different leaf traits (i.e., attributes, morphology, or texture) on the biochemical demand for photoprotection is shown in Figure 2. Foliar hairs efficiently decreased the biochemical demand for photoprotection (i.e., lower VAZ, α-Toc, and tAsc) even if they were present at the abaxial side of the leaf only. Higher requirement of biochemical photoprotection was found in evergreen than in deciduous leaves (this was remarkably evident in the higher contents of VAZ, α-Toc, and tAsc) and in summer-deciduous than in winter-deciduous leaves (evidenced in significantly higher Lut and tAsc, and higher, although not significantly, β-Car and AZ/VAZ). Besides having strongly developed structural photoprotective mechanisms, sclerophyllous leaves showed higher values of antioxidants L, VAZ, α-Toc, and tAsc than membranous leaves (Figure 2).

Associated to this, the predawn content of carotenoids and antioxidants (expressed in mmol mol^−1^ Chl) increased with leaf mass per area (LMA) (Figure 3). This relationship was significant for all the metabolites (including tGSH, which was represented by a low number of cases: *n* = 6), except for α-Toc. At lesser extent, Chl content also followed a similar trend, although the correlation was no significant (*P* > 0.05). No correlation at all was found between Chl a/b ratio and LMA, or between AZ/VAZ and LMA (Figure 3).

### Response to mediterranean climatic stressors

The most characteristic climatic stresses in Mediterranean areas, and as a consequence, the stresses more deeply studied in the literature were: LT, HT, D, and seasonality. Variation of the main photosynthetic pigments and antioxidants (by comparison with controls) in response to Mediterranean climatic stressors are shown in Figure 4. The results show that these stresses provoked an enhancement in the content of all the parameters, with the exception of Chl a+b under HT (Figure 4). Drought significantly enhanced Neo, Lut, β-Car, VAZ, AZ/VAZ and it was the stress that more intensively triggered the accumulation of α-Toc (Figure 4C). Low temperature increased the Chl a/b ratio, and Lut, β-Car and α-Toc contents, while HT enhanced the total VAZ pool, AZ/VAZ, Chl a/b, and α-Toc, and induced a significant decrease in Chl a+b. Seasonality provoked an augmentation in the contents of Neo, Chl a/b, β-Car, AZ/VAZ, and α-Toc (Figure 4). Interestingly, the total VAZ and the antioxidant α-Toc (Figures 4B,C) showed more responsiveness to all the stresses than the other parameters, being the amplitude of the change higher than in the other compounds. The hydrophilic antioxidants tGSH and tASC did not show any significant change under any of the climatic stressors (Figure 4C).

### Overnight retention of AZ/VAZ in mediterranean species

**Figure 5:**
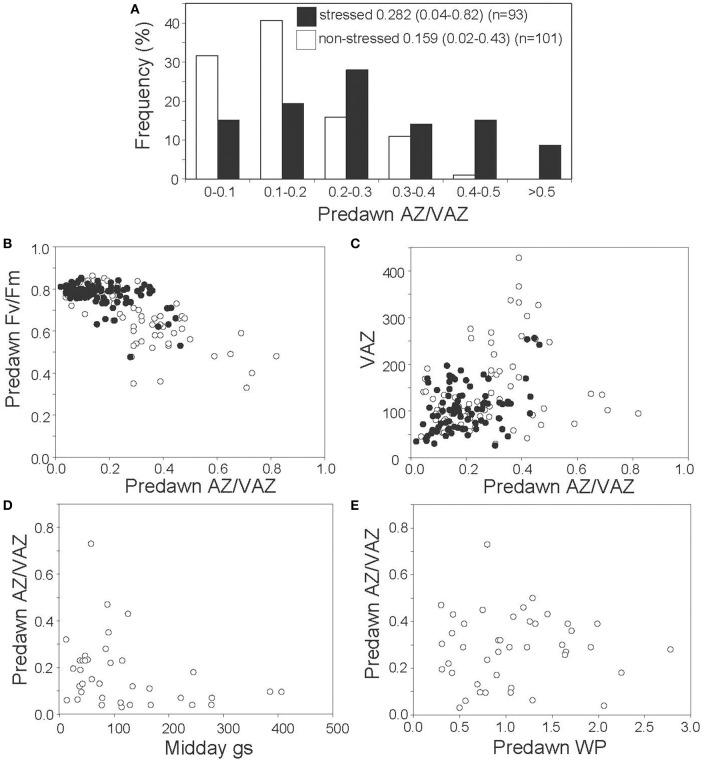
Nocturnal retention of AZ/VAZ in Mediterranean species. **(A)** Frequency histograms of the predawn AZ/VAZ in non-stressed (empty bars) and stressed (black bars) plants. Numbers denote the mean, the minimum and maximum values (in parenthesis) and the number of data. **(B)** Relationship between predawn AZ/VAZ and predawn Fv/Fm in winter (white circles) and other seasons (black circles). **(C)** Relationship between predawn AZ/VAZ (mol mol^−1^) and predawn VAZ pool (mol mol^−1^Chl) in winter (white circles) and in other seasons (black circles). **(D)** Relationship between predawn AZ/VAZ and midday stomatal conductance (g_*s*_ in mmol H_2_O m^−2^ s^−1^). **(E)** Relationship between predawn AZ/VAZ and predawn water potential (in −MPa).

To tackle the question of whether A+Z can be retained overnight under particular stress conditions in Mediterranean plants, frequencies of predawn values of AZ/VAZ in non-stressed and stressed plants were compared (Figure 5A). Predawn AZ/VAZ values higher than 0.4 were reported for 24% of stressed plants while only 1% of the non-stressed plants gave values higher than this threshold. Furthermore, photochemical efficiency (measured as predawn Fv/Fm) decreased with increasing overnight retention of Ant+Zea (measured as AZ/VAZ) (Figure 5B). This effect was particularly noticeable in winter, and to a lesser extent in other seasons, and correlated with the size of the VAZ pool (Figure 5C). However, apart from winter low temperatures, water stress also triggered the sustained retention of Ant+Zea (Figure 5D). Accordingly, data shown in Figure 5D evidenced that to attain high values of predawn AZ/VAZ, a low g_s_ is required and conversely, at high g_s_, overnight Ant+Zea is always low. Relationship between water potential (ψ) and predawn AZ/VAZ (Figure 5E) showed the same trend but less markedly.

### Content-plasticity of photosynthetic pigments and antioxidants

In order to assess the response capacity of main photosynthetic pigments and antioxidants in Mediterranean plants, a Plasticity Index (PI) was calculated for each of those parameters (Table 2). Higher plasticity was found in antioxidants than in pigment contents. Low variability in pigment content was generally found in Chl a+b, Chl a/b, Neo and β-Car. Among pigments, only VAZ and AZ/VAZ showed PI values above 0.5. Among antioxidants α-Toc showed higher PI than tAsc and tGSH suggesting a functional and important role for this metabolite in response to fluctuations in the environment.

**Table 2:**
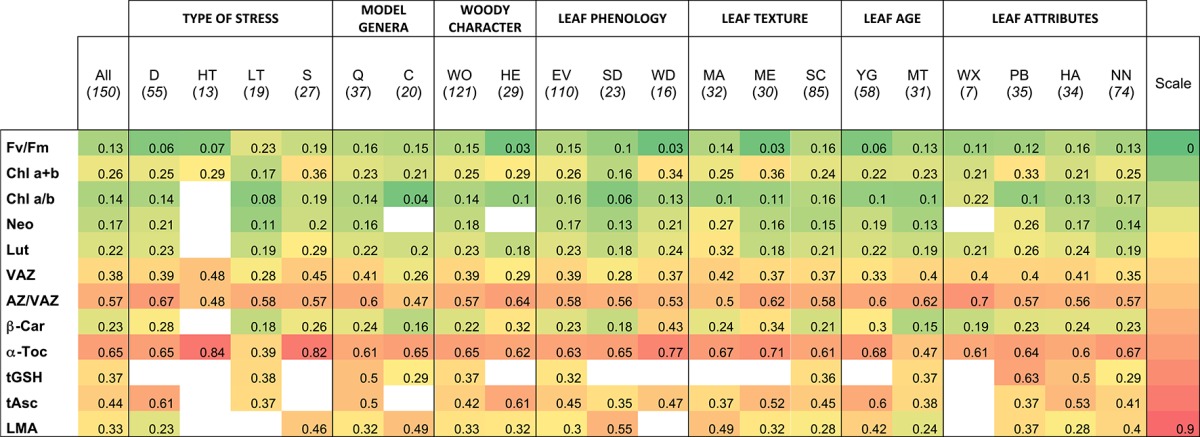
Plasticity index of main parameters according to relevant factors of variation considered.

*Units as in Figure 1. Low values appear in green and they get redder as they increase (see scale). Gaps indicate insufficient data to be calculated. D, Drought; HT, high temperature; LT, low temperature; S, seasonal; Q, Quercus; C, Cistus; WO, woody; HE, herb; EV, evergreen; SD, summer deciduous; WD, winter deciduous; MA, malacophyllous; ME, membranous; SC, sclerophyllous; YG, young; MT, mature; WX, Waxy; PB, pubescent, HA, hairy abaxial; NN, none. Number of cases (n) is indicated in brackets*.

### Sclerophyllous *Quercus* species as model mediterranean plants

**Figure 6:**
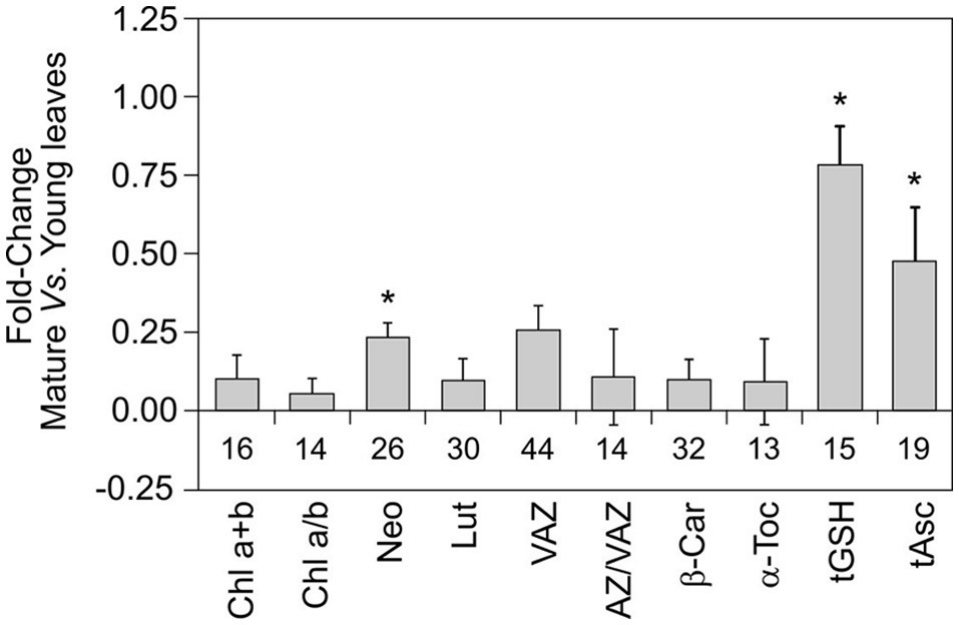
Fold-changes of photosynthetic pigments and antioxidants between mature and young leaves in sclerophyllous *Quercus* (*Q. coccifera*; *Q. ilex*; *Q. suber*). Only articles that studied jointly mature and young leaves were considered (total articles = 8). The data of young leaves were taken as reference values in each unit of the analyzed metabolites. The fold-changes were calculated as follows: Fold-change = (value of mature leave/value of young leave) −1. Thus, reference value was equaled to zero. Bar represents the mean ± SE. The number of reported data (*n*) is shown below each bar. Asterisk represent statistical significance between young and mature leaves (*P* < 0.05).

Within the 73 total species included in this study, higher number of cases concerned sclerophyllous *Quercus*, which is actually one of the most preponderant tree and an important model genus within the Mediterranean Basin ecosystems. The sclerophyllous leaves from oak contain relatively high levels of Chl a+b per area, when compared to other Mediterranean species (Table 1). The rest of photosynthetic pigments, (i.e., carotenoids per Chl ratios) however, followed a quite stable stoichiometry, being similar for most of the studied species (Table 1). Besides the well-known phenotypic/morphological plasticity of oak leaves, biochemical plasticity appeared also higher than in other species, e.g., higher plasticity indexes were obtained for Chl a/b, AZ/VAZ, α-Toc, and tGSH in *Quercus* than in the other well represented Mediterranean genus *Cistus* (Table 2). Regarding leaf age and development, mature leaves of sclerophyllous *Quercus* species had significantly higher content of all photosynthetic pigments and antioxidants (specially, Neo, tGSH, and tAsc) than young leaves (Figure 6). This fact, contrasts with the different influence of plant age over the antioxidant pool: i.e., VAZ and AZ/VAZ were lower in older than in younger plants, but higher in mature than in young leaves (Figure 1 vs. Figure 6).

## Discussion

### Interaction of morphological traits and photoprotection

Mediterranean-type ecosystems are characterized by a period of a summer drought, extreme temperatures, both in winter and summer, and a marked seasonality. This type of climate can be found in all continents, but the largest area is the Mediterranean basin. This biome, one of the richest in terms of plant diversity with 25,000 native species, is recognized as a Global Biodiversity Hotspot (Myers et al., 2000). The succession of these stresses requires the acclimation of plant species living in this ecosystem. In this sense, the plant photoprotective mechanisms are key strategies in the resilience of Mediterranean species to the harsh environment. Such is the case of evergreens with long lived leaves, which compared to deciduous are more likely to suffer and cope with more stressful conditions during their life duration. As expected, the morphological and biochemical characteristics should them adjust along seasons and years. For this reason, evergreens require more rigid mesophyll structure (Onoda et al., 2017) or greater investment in photoprotection (with higher contents of VAZ and α-Toc) than deciduous, as we shown in this study (Figure 2). Sclerophyllous leaves, a trait that is closely related to evergreen strategy, also showed the same pattern.

Leaf pubescence (presence of trichomes) and glaucescence (waxes) are two of the most conspicuous foliar traits in Mediterranean plants, giving these ecosystems one of their signs of identity. These structures represent an example of evolutionary convergence. They reduce water loss (Peguero-Pina et al., 2017) and they may be highly reflective, limiting the penetration of light to the photosynthetic apparatus and reaching the mesophyll (Agrawal et al., 2009; Camarero et al., 2012). Thereby they reduce the need of photoprotective pigments and antioxidants, supporting the photoprotective role of leaf pubescence in Mediterranean species (Morales et al., 2002). Alternatively, trichomes and waxes can act as protective response since their accumulation is triggered after extenuation of biochemical photoprotection, as has been evidence for the waxes in *Juniperus thurifera*, where their accumulation represented a symptom of species decline (Esteban et al., 2014). Here, we show that biochemical traits (antioxidants and pigments) compensate the absence of morphological protective traits in Mediterranean species (Figure 2).

Another characteristic of Mediterranean flora is, compared to other temperate biomes, the higher leaf thickness (Peguero-Pina et al., 2012) and LMA (Traiser et al., 2005). Despite structural filtering of light and efficient protection against water lost of most sclerophyllous leaves, it has been proposed the existence of a trade-off between photosynthesis and LMA in relation with Leaf Economics Spectrum (Wright et al., 2004). It seems that mesophyll conductance (g_s_) may be the most limiting factor for carbon assimilation, showing those leaves with highest LMA values, a reduction in CO_2_ diffusion to the carboxylation sites (Flexas et al., 2014; Onoda et al., 2017; Peguero-Pina et al., 2017). This implies that plants with high LMA will generally show lower photosynthetic capacity and thus, may require higher levels of photoprotective metabolites. Interestingly, we found a positive relationship between LMA and photoprotective compounds, confirming that thicker leaves had more photoprotective demand (Figure 3). The cost associated with greater investment in photoprotection compounds in high LMA leaves underpin longer lifespan in these species (Wright et al., 2004; Díaz et al., 2015), meaning that long-lasting leaves may cope with higher cumulative number of stressful events along their life-span.

### Pigments and photoprotection

Pigment composition in Mediterranean plants does not differ substantially from the standard stoichiometry of higher plants of other biomes. Thus, with a few exceptions, photosynthetic pigment composition of non-stressed Mediterranean plants (Table 1) fitted within the 95% percentile of the control values reported by Esteban et al. (2015a) in a wider survey, including more than 800 plant species from diverse biomes. Most of these exceptions were found in the genera *Quercus* and *Cistus*, with the high Chl a/b and β-Car values being singularly frequent. Stress responses diverged from some of the general patterns described by Esteban et al. (2015a) in intensity and direction. In general, most stress factors induced increases on protective carotenoid pools (Figures 4A,B) such as β-Car (Munné-Bosch et al., 2003), VAZ and Lut (Li et al., 2009), but also of structural components such as Neo (Figure 4A) that are supposed to be strongly stable (Schmid, 2008). Among the environmental stressors, drought was the factor that affected more strongly pigment composition followed by seasonality and low temperatures. This corresponds with the Mediterranean dual stress model, in which summer drought and, in some places winter low temperatures, determines plant responses and distribution (Mitrakos, 1982). Besides, a high degree of plasticity was observed in all these responses (Table 2). This is in agreement with the large phenotypic plasticity as one of the typical strategies of Mediterranean plants to cope with their changing environmental conditions (Hormaetxe et al., 2007).

Under severe stress conditions, specially low temperature, the VAZ cycle does not reach a complete relaxation (Demmig-Adams and Adams, 1996; Demmig-Adams et al., 2006), and AZ/VAZ is typically higher than 0.5 before dawn (Míguez et al., 2015). This process has been characterized in detail in boreal conifers and alpine evergreens (Verhoeven, 2014; Míguez et al., 2017) involving profound change in the organization of photosynthetic apparatus. The overnight retention of AZ/VAZ is usually concurrent with a proportional decrease of photochemical efficiency (measured as Fv/Fm) (Demmig-Adams et al., 2006), also known as “winter photoinhibition.” In Mediterranean evergreens, the existence of an inverse relationship between AZ/VAZ and Fv/Fm, notably in winter (Figure 5B), suggests that Mediterranean evergreens employ the same mechanism of down-regulation of photochemical efficiency as boreal and alpine evergreens. Furthermore, present analysis suggests that this relationship extends to other stress factors rather than low temperature (Figure 5B, white dots), particularly water stress, which is the main environmental driver in Mediterranean ecosystems. Thus, high predawn AZ/VAZ values were observed only in plants with a low stomatal conductance (Figure 5D). Drought-stress induction of overnight retention of A+Z has been described in desert and desiccation-tolerant plants (Barker et al., 2002; Fernández-Marín et al., 2011, 2013). Here, we confirm that this mechanism is also activated at milder stress levels, such as that exerted by Mediterranean summer, being more universal than previously expected. Nevertheless, the high dispersion of values shown in Figure 5 indicates that the retention of A+Z does not always ensure a decrease in photochemical efficiency, likely playing other roles in the responses to stress (Havaux and Niyogi, 1999; Dall'Osto et al., 2010).

### Role of lipophilic and hydrophilic antioxidants

In contrast to the constricted stoichiometry of pigment composition in chloroplasts caused by the binding of pigments to thylakoid proteins (Antal et al., 2013), antioxidants display a much higher degree of variation, as we show in this study (Table 1). Although all species have available the same set of low molecular weight lipophilic (α-Toc, β-Car, and Zea) and hydrophilic antioxidants (Asc and GSH) to cope with oxidative stress, strong differences exist depending on taxonomy, plant growth and leaf characteristics (phenology, texture, age, attributes) (Figures 1, 2) and in response to stressful conditions (Figure 4). Thus, such differences can reach several orders of magnitude for α-Toc and tAsc (Table 1, Figures 1, 4). Curiously, despite Asc is the most abundant antioxidant in leaves (Table 1), neither tAsc nor tGSH was induced by any climatic stressors (Figure 4). This result suggests that most photo-oxidative damage undergone by Mediterranean species comes from the chloroplast, where α-Toc and β-Car are embedded in thylakoid membranes (Antal et al., 2013).

Interestingly, α-Toc and VAZ contents in angiosperms significantly differed from those found in gymnosperms (Figure 2). In angiosperms, there was a higher α-Toc and a lower VAZ contents than in gymnosperm. Within angiosperm, the higher content of α-Toc in woody species suggests an increased demand for α-Toc associated to the woody trait. This result is verified by an increment of α-Toc from seedling to adults, showing at the same time a decrease in VAZ (Figure 1). As stated before by Esteban et al. (2009), α-Toc increases progressively in the phylogeny from green algae to gymnosperms and even more to angiosperms, whereas the size of total VAZ pool shows the opposite pattern. This supports the idea that VAZ pigments and α-Toc share some photoprotective functions, which may lead to some degree of inter-compensation (Havaux and García-Plazaola, 2014). The cooperative function of VAZ and α-Toc is accounted because both are lipophilic compounds synthesized in the plastids being able to interact with membranes where they share several functions. Both are antioxidants (Falk and Munne-Bosch, 2010) and quenchers of ^1^O_2_ (Triantaphylidès and Havaux, 2009) and carotenoids may act also as membrane stabilizers (Havaux, 1998) and as quenchers of triplet Chl (Esteban et al., 2015b). Consequently, in agreement with Wujeska et al. (2013) VAZ and α-Toc exhibit the highest plasticity among pigments and antioxidants, respectively (Table 2). The paramount importance of α-Toc in the photoprotective response of Mediterranean plants is confirmed by the fact that it was the parameter that showed the highest responsiveness under all stresses analyzed in this manuscript (Figure 4 and Table 2). Among stressors, drought induced the highest α-Toc increase, followed by low and high temperatures, highlighting the importance of climatic stressors on the performance of plants in the Mediterranean ecosystems.

### Concluding remarks

Throughout this work several gaps of knowledge related to photoprotection in Mediterranean plants have been identified: (i) herbs have been much less studied than woody species, (ii) and gymnosperms than angiosperms; and (iii) much less data are available about hydrophilic antioxidants than about lipophilic ones. Despite of these limitations in the literature, several conclusive statements have arisen from this analysis. The traits woody and high LMA, were generally related with a high demand for photoprotection. This can be explained by the evergreen strategy of most Mediterranean plants that implies high need of protection because of the long lifespan of their leaves. tGSH and tAsc showed the highest amplitude in their content. Nevertheless, α-Toc and VAZ were more responsive against Mediterranean climatic stressors. Thus, α-Toc responded mainly to drought and also to high temperature, while VAZ responded mainly to low temperature and also to drought. Therefore, the powerful photoprotective system of Mediterranean species pivots on lipophilic antioxidants, in particular Z and α-Toc, which play multiple roles in the plastid. In the context of a global climatic change, in which drought and extreme temperatures events are foreseen to increase their frequency in the Mediterranean area, the plasticity of photoprotective mechanisms to cope with such enhancing stressors will be fundamental. Resilience of Mediterranean species, compared with the template flora will likely benefit from such higher plasticity. To fully address these questions a complete understanding of this issue covering ecoregions other than the Mediterranean Basin (e.g., Western and South Australia, Cape Region, coastal California, and Chile) is needed.

## Author contributions

All authors listed have made a substantial, direct and intellectual contribution to the work, and approved it for publication.

### Conflict of interest statement

The authors declare that the research was conducted in the absence of any commercial or financial relationships that could be construed as a potential conflict of interest. The reviewer LLVJ and handling Editor declared their shared affiliation, and the handling Editor states that the process nevertheless met the standards of a fair and objective review

## Abbreviations

Ant: anteraxanthin
α-Toc: α-tocopherol
AZ/VAZ: de-epoxidation state of the violaxanthin cycle
β-Car: β-carotene
*C*: *Cistus*
Chl: chlorophyll
D: drought
DW: dry weight
EV: evergreen
Fv/Fm: maximal photochemical efficiency of PSII
FW: fresh weight
g_m_: mesophyll conductance
g_s_: stomatal conductance
HA: hairy abaxial
HE: herb
HL: high light
HT: high temperature
LMA: leaf mass per area
LT: low temperature
Lut: lutein
Lx: lutein epoxide
Lx-cycle: lutein-epoxide cycle
MA: malacophyllous
ME: membranous
MT: mature
Neo: neoxanthin
NN: none
^1^O_2_: singlet oxygen
PI: plasticity index
PB: pubescent
PSII: photosystem II
*Q*: *Quercus*
ROS: reactive oxygen species
S: seasonality
SC: sclerophyllous
SD: summer deciduous
tAsc: total ascorbic acid
tGSH: total glutathione
VAZ-cycle: violaxanthin cycle
Vio: violaxanthin
Ψ: water potential
WD: winter deciduous
WO: woody
WUE: water use efficiency
WX: Waxy
YG: young
Zea: zeaxanthin.
