# Supplementary material for: Photoprotective Strategies of Mediterranean Plants in Relation to Morphological Traits and Natural Environmental Pressure: A Meta-Analytical Approach
Source: Front Plant Sci. 2017 Jun 19;8:1051. doi: 10.3389/fpls.2017.01051 (PMC5474485; doi:10.3389/fpls.2017.01051)
Supplement: Supplementary file 1 [file Table1.DOCX]

**SUPPLEMENTARY MATERIAL**

**Table S1. Complete list of families and species included in this work.**

| Family | Species |
| --- | --- |
| Aceraceae | *Acer monspessulanum* |
| Anacardiaceae | *Pistacia lentiscus* |
|  | *Pistacia terebinthus* |
| Apiaceae | *Apium nodiflorum* |
|  | *Foeniculum vulgare* |
| Aquifoliaceae | *Ilex aquifolium* |
| Asteraceae | *Chondrilla juncea* |
|  | *Cichorium intybus* |
|  | *Scolymus hispanicus* |
|  | *Silybum marianum* |
|  | *Taraxacum obovatum* |
| Brassicaceae | *Diplotaxis ibicensis* |
| Buxaceae | *Buxus sempervirens* |
| Cannabaceae | *Humulus lupulus* |
| Capparaceae | *Capparis spinosa* |
| Caprifoliaceae | *Lonicera implexa* |
|  | *Viburnum lantana* |
|  | *Viburnum tinus* |
| Caryophyllaceae | *Silene vulgaris* |
| Chenopodiaceae | *Beta maritima* |
|  | *Beta maritime marcusii* |
|  | *Beta maritime maritima* |
| Cistaceae | *Cistus albidus* |
|  | *Cistus clusii* |
|  | *Cistus creticus* |
|  | *Cistus ladanifer* |
|  | *Cistus monspeliensis* |
|  | *Cistus salviifolius* |
|  | *Halimium halimifolium* |
| Cupressaceae | *Cupressus lusitanica* |
|  | *Juniperus phoenicea* |
|  | *Juniperus thurifera* |
| Curcubitaceae | *Bryonia dioica* |
| Dioscoreaceae | *Tamus communis* |
| Ericaceae | *Arbutus unedo* |
|  | *Arctostaphyll usuva-ursi* |
|  | *Erica multiflora* |
|  | *Erica scoparia* |
| Fagaceae | *Quercus cerris* |
|  | *Quercus coccifera* |
|  | *Quercus frainetto* |
|  | *Quercus humilis* |
|  | *Quercus ilex* |
|  | *Quercus suber* |
| Hypericaceae | *Hypericum balearicum* |
| Lamiaceae | *Lavandula stoechas* |
|  | *Melissa officinalis* |
|  | *Phlomis italica* |
|  | *Rosmarinus officinalis* |
|  | *Salvia lanigera* |
|  | *Salvia officinalis* |
| Lauraceae | *Laurus nobilis* |
| Liliaceae | *Allium ampeloprasum* |
|  | *Asparagus acutifolius* |
| Malvaceae | *Lavathera maritima* |
| Montiaceae | *Montia fontana* |
| Myrtaceae | *Eucaliptus globulus* |
|  | *Eucalyptus gunnii* |
|  | *Myrtus communis* |
| Oleaceae | *Arbutus unedo* |
|  | *Olea europaea* |
|  | *Phyllirea angustifolia* |
|  | *Phyllirea latifolia* |
| Papaveraceae | *Papaver rhoeas* |
| Pinaceae | *Pinus halepensis* |
|  | *Pinus pinea* |
| Plumbaginaceae | *Limonium gibertii* |
|  | *Limonium magallufianum* |
| Polygonaceae | *Rumex papillaris* |
|  | *Rumex pulcher* |
| Rhamnaceae | *Rhamnus alaternus* |
|  | *Rhamnus ludovici-salvatoris* |
| Rosaceae | *Prunus cerasus* |
