# Supplementary material for: Photoprotective Strategies of Mediterranean Plants in Relation to Morphological Traits and Natural Environmental Pressure: A Meta-Analytical Approach
Source: Front Plant Sci. 2017 Jun 19;8:1051. doi: 10.3389/fpls.2017.01051 (PMC5474485; doi:10.3389/fpls.2017.01051)
Supplement: Supplementary file 2 [file Table2.DOCX]

**SUPPLEMENTARY MATERIAL**

**Table S2. Complete list of manuscripts used for data compilation.**

| **1^st^ author** | **Year** | **Journal** | **Vol** | **1^st^ page** |
| --- | --- | --- | --- | --- |
| Abreu. ME | 2007 | Physiologia Plantarum | 131 | 590 |
| Abreu. ME | 2008 | Environmental and Experimental Botany | 64 | 105 |
| Ain-Lhout. F | 2004 | Photosynthetica | 42 | 399 |
| Balaguer. L | 2001 | Functional Ecology | 15 | 124 |
| Baraldi. R | 2008 | Photosynthetica | 46 | 378 |
| Bartoli. G | 2014 | ComptesRendusBiologies | 337 | 101 |
| Brossa. R | 2015 | Planta | 241 | 4 |
| Camarero. JJ | 2012 | Flora | 207 | 557 |
| Corcuera. L | 2005 | Tree Physiology | 25 | 599 |
| Corcuera. L | 2005 | Trees-Structure and function | 19 | 99 |
| Martínez-Ferri. E | 2000 | Tree Physiology | 20 | 131 |
| Esteban. R | 2014 | Tree Physiology | 34 | 267 |
| Faria. T | 1996 | Tree Physiology | 16 | 115 |
| Faria. T | 1998 | Physiologia Plantarum | 102 | 419 |
| Fleck.I | 2003 | Plant Physiology and Biochemistry | 41 | 41 |
| Fleck. I | 2000 | Australian Journal of Plant Physiology | 27 | 129 |
| Galmes. J | 2007 | Physiologia Plantarum | 130 | 495 |
| Garcia-Plazaola. JI | 2003 | Trees | 17 | 285 |
| Garcia-Plazaola. JI | 1999 | Plant Science | 143 | 125 |
| Garcia-Plazaola. JI | 2000 | Plant Biology | 2 | 530 |
| GarciaPlazaola. JI | 2008 | Trees-Structure and Function | 22 | 385 |
| GarciaPlazaola. JI | 1999 | Journal of plant physiology | 155 | 625 |
| GarciaPlazaola. JI | 1997 | Journal of Experimental Botany | 48 | 1667 |
| Grant. OM | 2015 | Functional Plant Biology | 42 | 219 |
| Guimaraes. R | 2009 | Industrial Crops and Products | 30 | 427 |
| Gulias. J | 2002 | Tree Physiology | 22 | 687 |
| Hernandez. I | 2004 | Tree Physiology | 24 | 1303 |
| Hormaetxe. K | 2004 | Plant Biology | 6 | 325 |
| Nogues. I | 2012 | Plant Ecology | 213 | 649 |
| Peguero-Pina. JJ | 2013 | Journal of Experimental Botany | 64 | 1649 |
| Jubany-Mari. T | 2009 | Journal of Experimental Botany | 60 | 107 |
| Jubany-Mari. T | 2010 | Environmental and Experimental Botany | 69 | 47 |
| Juvany. M | 2012 | Journal of Integrative Plant Biology | 54 | 584 |
| Juvany. M | 2013 | Journal of Experimental Botany | 65 | 1039 |
| Kytridis. VP | 2008 | Journal of Plant Physiology | 165 | 952 |
| Levizou. E | 2004 | Photosynthetica | 42 | 229 |
| Llorens. L | 2002 | Functional Plant Biology | 29 | 81 |
| Llusia. J | 2005 | Physiologia Plantarum | 123 | 40 |
| Manetas. Y | 2003 | Functional Plant Biology | 30 | 265 |
| Manetas. Y | 2004 | Photosynthetica | 42 | 591 |
|  |  |  |  |  |
|  |  |  |  |  |
|  |  |  |  |  |
| **1^st^ author** | **Year** | **Journal** | **Vol** | **1^st^ page** |
| Martinez-Ferri. E | 2002 | Trees-Structure and Function | 16 | 504 |
| Morales. F | 2002 | Trees-Structure and Function | 16 | 504 |
| Muller. M | 2006 | Journal of Plant Physiology | 163 | 601 |
| Munne-Bosch. S | 2001 | Journal of Plant Physiology | 158 | 1431 |
| Munne-Bosch. S | 2001 | Plant Cell and Environment | 24 | 1319 |
| Munne-Bosch. S | 2002 | Planta | 214 | 608 |
| Munne-Bosch. S | 2003 | Annals of Botany | 92 | 385 |
| Munne-Bosch. S | 2003 | Plant Physiology | 131 | 1816 |
| Munne-Bosch. S | 2003 | Planta | 217 | 758 |
| Munne-Bosch. S | 2003 | Tree Physiology | 23 | 1 |
| Munne-Bosch. S | 2004 | New Phytologist | 162 | 115 |
| Munne-Bosch. S | 2004 | Plant Science | 166 | 1105 |
| Munne-Bosch. S | 2000 | Australian Journal of Plant Physiology | 27 | 139 |
| Munne-Bosch. S | 2000 | PhysiologiaPlantarum | 108 | 147 |
| Munne-Bosch. S | 2000 | Planta | 210 | 925 |
| Munne-Bosch. S | 2001 | Australian Journal of Plant Physiology | 28 | 315 |
| Munne-Bosch. S | 2009 | Journal of Plant Physiology | 166 | 136 |
| Munne-Bosch. S | 2007 | Planta | 225 | 1039 |
| Nogues. I | 2015 | Environmental and experimental botany | 119 | 76 |
| Nogues. I | 2014 | Plant Biosystems | 148 | 268 |
| Olivan. A | 2010 | Journal of Arid Environments | 74 | 1572 |
| Morales. P | 2012 | Genet Resour Crop Evol | 59 | 851 |
| Peguero-Pina. JJ | 2008 | Oecologia | 156 | 1 |
| Rubio De Casas. R | 2007 | Annals of Botany | 100 | 325 |
| Sánchez-Mata. MC | 2012 | Genetic Resources and Crop Evolution | 59 | 431 |
| Silva-Cancino. MC | 2012 | PhysiologiaPlantarum | 144 | 289 |
| Tattini. M | 2009 | Functional Plant Biology | 36 | 551 |
| Tlili. N | 2010 | Plant Foods for Human Nutrition | 65 | 260 |
| Tlili. N | 2009 | Journal of Agricultural and Food Chemistry | 57 | 5381 |
| Tounekti. T | 2011 | Environmental & Experimental Botany | 71 | 298 |
| Valladares. F | 2000 | New Phytologist | 148 | 79 |
| Wolkerstorfer. SV | 2011 | Trees-Structure and function | 25 | 1043 |
