# Supplementary material for: Photoprotective Strategies of Mediterranean Plants in Relation to Morphological Traits and Natural Environmental Pressure: A Meta-Analytical Approach
Source: Front Plant Sci. 2017 Jun 19;8:1051. doi: 10.3389/fpls.2017.01051 (PMC5474485; doi:10.3389/fpls.2017.01051)
Supplement: Supplementary file 3 [file Table3.DOCX]

**SUPPLEMENTARY MATERIAL**

**Table S3 Composition of photosynthetic pigment and low molecular weight antioxidants in a representation of wild Mediterranean species.** Data are mean, maximum and minimum for each species and metabolite. Number of cases is designated as *n*. Species for which number of cases of at least 2 was available are shown: i.e. *n*≥2. Units as in Figure 1.

| Family | *Species* | Chl a+b | | | | Chl a/b | | | | Neo | | | | Lut | | | | VAZ | | | |
| --- | --- | --- | --- | --- | --- | --- | --- | --- | --- | --- | --- | --- | --- | --- | --- | --- | --- | --- | --- | --- | --- |
|  |  | mean | max | min | n | mean | max | min | n | mean | max | min | n | mean | max | min | n | mean | max | min | n |
| Anacardiaceae | *Pistacia lentiscus* | **344** | 379 | 288 | 5 | **2.46** | 2.94 | 1.79 | 5 |  |  |  |  | **123** | 127 | 120 | 2 | **86** | 135 | 52 | 3 |
| Buxaceae | *Buxus sempervirens* | **321** | 546 | 257 | 7 | **2.22** | 3.72 | 1.75 | 5 | **36** | 41 | 32 | 3 | **159** | 188 | 121 | 6 | **112** | 153 | 35 | 7 |
| Caprifoliaceae | *Lonicera implexa* | **305** | 336 | 262 | 3 |  |  |  |  |  |  |  |  | **124** | 126 | 123 | 2 | **86** | 98 | 66 | 3 |
|  | *Viburnum tinus* | **377** | 395 | 356 | 3 |  |  |  |  |  |  |  |  | **158** | 163 | 154 | 2 | **106** | 129 | 73 | 3 |
| Cistaceae | *Cistus albidus* | **303** | 467 | 151 | 5 | **3.03** | 3.73 | 2.65 | 3 | **39** | 47 | 30 | 2 | **132** | 186 | 93 | 4 | **56** | 94 | 30 | 4 |
|  | *Cistus clusii* | **348** | 375 | 311 | 3 | **2.78** | 2.85 | 2.67 | 6 | **48** | 52 | 45 | 4 | **289** | 543 | 200 | 5 | **91** | 101 | 75 | 4 |
|  | *Cistus salviifolius* | **476** | 625 | 370 | 4 | **3.25** | 3.25 | 3.25 | 1 | **33** | 35 | 31 | 2 | **118** | 120 | 114 | 3 | **52** | 57 | 47 | 3 |
|  | *Halimium halimifolium* |  |  |  |  | **3.23** | 3.43 | 3.02 | 2 |  |  |  |  |  |  |  |  |  |  |  |  |
| Cupressaceae | *Juniperus phoenicea* | **359** | 382 | 336 | 2 |  |  |  |  |  |  |  |  |  |  |  |  | **147** | 161 | 132 | 2 |
|  | *Juniperus thurifera* |  |  |  |  |  |  |  |  |  |  |  |  | **149** | 150 | 149 | 2 | **107** | 107 | 107 | 2 |
| Ericaceae | *Arbutus unedo* | 407 | 561 | 178 | 5 | 3.10 | 3.44 | 2.76 | 2 | 46 | 58 | 33 | 2 | 116 | 124 | 106 | 3 | 85 | 134 | 62 | 4 |
|  | *Erica multiflora* |  |  |  |  | **2.59** | 3.10 | 2.08 | 2 |  |  |  |  | **441** | 683 | 199 | 2 | **263** | 370 | 155 | 2 |
|  | *Quercus cerris* |  |  |  |  | **3.11** | 3.17 | 3.05 | 2 | **38** | 39 | 37 | 2 | **58** | 59 | 58 | 2 | **25** | 29 | 22 | 2 |
| Fagaceae | *Quercus coccifera* | **461** | 616 | 112 | 17 | **2.90** | 3.45 | 2.10 | 8 | **43** | 63 | 27 | 8 | **157** | 210 | 123 | 9 | **111** | 176 | 30 | 18 |
|  | *Quercus frainetto* |  |  |  |  | **3.54** | 3.66 | 3.42 | 2 | **52** | 56 | 48 | 2 | **65** | 72 | 57 | 2 | **34** | 35 | 33 | 2 |
|  | *Quercus ilex* | **528** | 791 | 304 | 19 | **3.20** | 4.07 | 1.97 | 18 | **36** | 56 | 26 | 18 | **142** | 218 | 104 | 19 | **85** | 197 | 40 | 25 |
|  | *Quercus suber* | **521** | 639 | 180 | 8 | **3.30** | 3.51 | 3.10 | 6 | **33** | 51 | 24 | 7 | **134** | 204 | 94 | 7 | **60** | 104 | 26 | 7 |
|  | *Melissa officinalis* | **160** | 238 | 83 | 2 | **2.44** | 2.48 | 2.40 | 2 |  |  |  |  | **186** | 186 | 186 | 1 | **88** | 88 | 88 | 1 |
| Lamiaceae | *Rosmarinus officinalis* | **278** | 408 | 117 | 3 | **2.96** | 3.12 | 2.80 | 3 | **44** | 44 | 43 | 2 | **270** | 412 | 196 | 3 | **47** | 67 | 9 | 3 |
|  | *Salvia officinalis* | **297** | 328 | 267 | 2 | **2.52** | 2.60 | 2.40 | 3 | **49** | 49 | 49 | 1 | **186** | 218 | 153 | 2 | **93** | 93 | 93 | 1 |
|  | *Olea europaea* |  |  |  |  |  |  |  |  |  |  |  |  |  |  |  |  | **143** | 167 | 118 | 2 |
| Oleaceae | *Phyllirea angustifolia* |  |  |  |  |  |  |  |  |  |  |  |  |  |  |  |  | **69** | 72 | 66 | 2 |
|  | *Phyllirea latifolia* | **487** | 583 | 381 | 3 |  |  |  |  |  |  |  |  | **137** | 141 | 133 | 2 | **114** | 182 | 89 | 4 |
|  | *Pinus halepensis* | **263** | 266 | 259 | 2 |  |  |  |  |  |  |  |  |  |  |  |  | **152** | 170 | 133 | 2 |
| Rhamnaceae | *Rhamnus alaternus* | **564** | 688 | 344 | 3 |  |  |  |  |  |  |  |  | **128** | 134 | 122 | 2 | **95** | 126 | 75 | 3 |

| Family | *Species* | AZ/VAZ | | | | b-Car | | | | a-Toc | | | | tGSH | | | | tAsc | | | |
| --- | --- | --- | --- | --- | --- | --- | --- | --- | --- | --- | --- | --- | --- | --- | --- | --- | --- | --- | --- | --- | --- |
|  |  | mean | max | min | n | mean | max | min | n | mean | max | min | n | mean | max | min | n | mean | max | min | n |
| Anacardiaceae | *Pistacia lentiscus* | **0.34** | 0.87 | 0.04 | 5 | **95** | 140 | 50 | 2 | **1599** | 2798 | 7 | 38 |  |  |  |  |  |  |  |  |
| Buxaceae | *Buxus sempervirens* | **0.18** | 0.36 | 0.02 | 7 | **86** | 97 | 68 | 6 | **1534** | 3914 | 69 | 5 | **680** | 789 | 571 | 2 | **23339** | 24025 | 22654 | 2 |
| Caprifoliaceae | *Lonicera implexa* | **0.16** | 0.21 | 0.11 | 3 | **86** | 89 | 83 | 2 | **260** | 414 | 63 | 3 | **318** | 326 | 310 | 2 | **4609** | 5730 | 3488 | 2 |
|  | *Viburnum tinus* | **0.16** | 0.21 | 0.11 | 3 | **96** | 96 | 96 | 2 | **745** | 1442 | 220 | 3 | **301** | 321 | 280 | 2 | **8041** | 9694 | 6388 | 2 |
| Cistaceae | *Cistus albidus* | **0.19** | 0.31 | 0.09 | 4 | **102** | 121 | 91 | 3 | **259** | 433 | 96 | 4 | **134** | 158 | 109 | 2 | **4056** | 4801 | 3310 | 2 |
|  | *Cistus clusii* | **0.44** | 0.50 | 0.33 | 4 | **185** | 397 | 126 | 5 | **153** | 344 | 36 | 8 |  |  |  |  | **18109** | 18109 | 18109 | 1 |
|  | *Cistus salviifolius* | **0.21** | 0.53 | 0.06 | 4 | **100** | 122 | 86 | 3 | **195** | 286 | 89 | 4 | **7** | 9 | 5 | 2 | **7390** | 8545 | 6236 | 2 |
|  | *Halimium halimifolium* |  |  |  |  | **152** | 155 | 149 | 2 |  |  |  |  |  |  |  |  |  |  |  |  |
| Cupressaceae | *Juniperus phoenicea* | **0.12** | 0.18 | 0.06 | 2 |  |  |  |  |  |  |  |  |  |  |  |  |  |  |  |  |
|  | *Juniperus thurifera* | **0.07** | 0.07 | 0.07 | 2 |  |  |  |  |  |  |  |  |  |  |  |  |  |  |  |  |
| Ericaceae | *Arbutus unedo* | 0.32 | 0.78 | 0.05 | 6 | 80 | 100 | 47 | 3 | 342 | 480 | 122 | 3 | 37 | 48 | 26 | 2 | 13459 | 14258 | 12660 | 2 |
|  | *Erica multiflora* |  |  |  |  | **250** | 380 | 121 | 2 |  |  |  |  |  |  |  |  | **2565** | 3603 | 1527 | 2 |
| Fagaceae | *Quercus cerris* |  |  |  |  | **78** | 80 | 77 | 2 | **126** | 155 | 98 | 2 |  |  |  |  | **2057** | 2119 | 1995 | 2 |
|  | *Quercus coccifera* | **0.23** | 0.49 | 0.09 | 17 | **110** | 173 | 31 | 8 | **469** | 606 | 212 | 3 | **30** | 31 | 28 | 2 | **12090** | 12102 | 12079 | 2 |
|  | *Quercus frainetto* |  |  |  |  | **98** | 106 | 90 | 2 | **75** | 76 | 73 | 2 |  |  |  |  | **2204** | 2687 | 1721 | 2 |
|  | *Quercus ilex* | **0.25** | 0.86 | 0.04 | 26 | **101** | 175 | 53 | 19 | **304** | 815 | 1 | 11 | **85** | 141 | 34 | 6 | **5762** | 13553 | 1335 | 7 |
|  | *Quercus suber* | **0.21** | 0.30 | 0.09 | 7 | **91** | 161 | 61 | 7 |  |  |  |  |  |  |  |  |  |  |  |  |
| Lamiaceae | *Melissa officinalis* | **0.15** | 0.18 | 0.12 | 2 | **120** | 120 | 120 | 1 | **158** | 212 | 105 | 2 |  |  |  |  |  |  |  |  |
|  | *Rosmarinus officinalis* | **0.23** | 0.29 | 0.13 | 3 | **136** | 218 | 83 | 5 | **52** | 156 | 4 | 5 |  |  |  |  |  |  |  |  |
|  | *Salvia officinalis* | **0.08** | 0.12 | 0.03 | 3 | **117** | 155 | 79 | 2 | **83** | 152 | 15 | 2 |  |  |  |  |  |  |  |  |
| Oleaceae | *Olea europaea* | **0.24** | 0.37 | 0.12 | 2 | **133** | 133 | 133 | 1 |  |  |  |  |  |  |  |  |  |  |  |  |
|  | *Phyllirea angustifolia* |  |  |  |  | **470** | 471 | 469 | 2 | **356** | 377 | 335 | 2 |  |  |  |  |  |  |  |  |
|  | *Phyllirea latifolia* | **0.19** | 0.37 | 0.09 | 4 | **97** | 100 | 94 | 2 | **288** | 416 | 109 | 3 | **127** | 140 | 115 | 2 | **8836** | 12950 | 4723 | 2 |
| Pinaceae | *Pinus halepensis* | **0.12** | 0.19 | 0.06 | 2 |  |  |  |  |  |  |  |  |  |  |  |  |  |  |  |  |
| Rhamnaceae | *Rhamnus alaternus* | **0.11** | 0.13 | 0.08 | 4 | **99** | 100 | 99 | 2 | **323** | 496 | 207 | 3 | **323** | 328 | 318 | 2 | **9084** | 9336 | 8832 | 2 |
